# Pneumoperitoneum after placement of the temporary pacing wires: transverse colon injury at the base of a diverticulum

**DOI:** 10.1007/s12055-024-01790-x

**Published:** 2024-08-02

**Authors:** Ivo Gasparovic, Panagiotis Artemiou, Stefan Durdik, Erika Drangova, Michal Hulman

**Affiliations:** 1 Faculty of Medicine, Clinic of Cardiac Surgery, Comenius University, National Institute of Cardiovascular Diseases, Pod Krasnou Hôrkou 1, 83101 Bratislava, Slovakia; 2 Faculty of Medicine, Clinic of Surgical Oncology, Comenius University, St. Elizabeth Oncology Institute, Bratislava, Slovakia; 3 Faculty of Medicine, Clinic of Radiology, Slovak Medical University, National Institute of Cardiovascular Diseases, Bratislava, Slovakia

**Keywords:** Pneumo-peritoneum, Epicardial electrodes placement, Transverse colon, Diverticulum injury

## Abstract

We present the successful management of a patient presenting with pneumo-peritoneum early after surgery due to transvere colon injury after placement of the temporary pacing wires. The patient was asymptomatic, underwent computed tomography, the temporary pacing wires were removed and he was managed conservatively.

## Case report

**Fig. 1:**
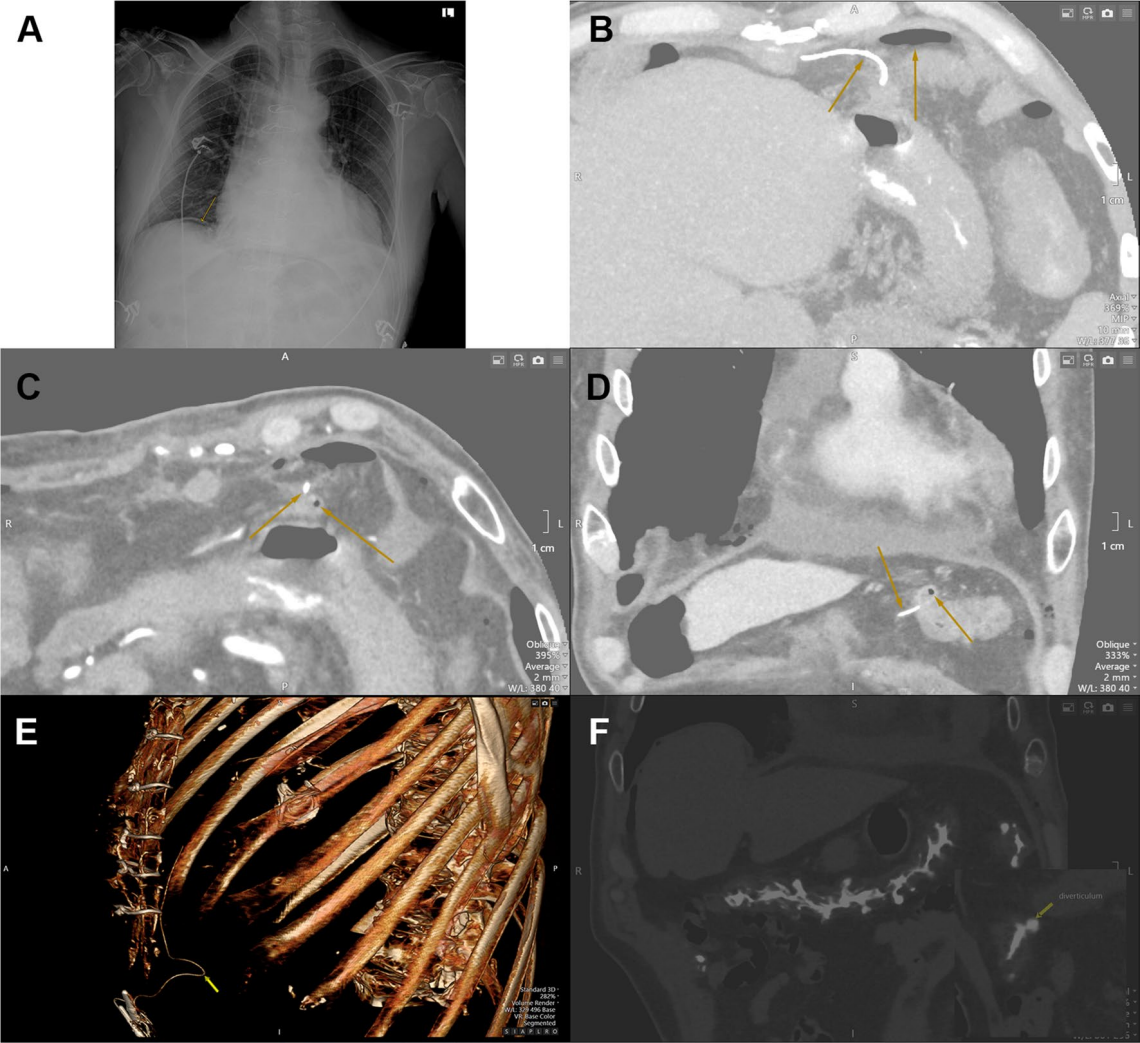
**A**, **B**: postoperative chest x-ray and computed tomography showing the pneumo-peritoneum **C**, **D**, **E**: computed tomography showing the injury of the transverse colon at the base of a diverticulum due to the needle end of temporary pacing wires passing through it **F **computed tomography showing the diverticulum of the transverse colon

A 75-year old male patient underwent a bio-Bentall procedure. On post-operative day 2 pneumo-peritoneum was diagnosed (Fig. 1A, B). Computed tomography (CT) showed injury of the transverse colon at the base of a diverticulum due to the needle end of temporary pacing wires passing through it, after placement of the epicardial temporary pacing wires (Fig. 1C, D, E), with no contrast leak (Fig. 1F). The epicardial temporary pacing wires were removed, the patient was asymptomatic, was managed conservatively and discharged home.

The patient signed an informed consent to present these images.

## Discussion

Pneumo-peritoneum is not a common finding in the immediate postoperative period [1]. Causes include, sub-diaphragmatic air entrance during drain placement, opening the peritoneal cavity during full median sternotomy, or perforation of an abdominal organ [2]. Pneumo-peritoneum, as a result of inadvertent opening of the peritoneal cavity during the initial surgical incision or during the subsequent cardiac surgery, is generally of no significance to the patient, although it must be distinguished from pneumo-peritoneum occuring secondary to intra-abdominal pathology [3]. Another cause of pneumo-peritoneum that has been described in literature was from intrathoracic pathology, like tension pneumothorax, and was treated percutaneously [4]. The patient didn´t have any symptoms of acute abdomen and during median sternotomy, or drain placement, the peritoneal cavity was not opened. CT excluded perforation of an abdominal organ and showed the transverese colon injury at the base of a diverticulum. Finally, transverse colon diverticulum is also a rare finding accounting for less than 6% of all gastrointestinal tract diverticula [5].

## Conclusion

CT has a central role in establishing the differential diagnosis in these patients, and in cases of colonic puncture, the patients can be treated conservatively with removal of the epicardial temporary pacing wires.

## Data Availability

The data are available on request from the corresponding author.
